# 2-Sulfanyl­idene-1,2-dihydro­pyridine-3-carbohydrazide

**DOI:** 10.1107/S160053681003521X

**Published:** 2010-09-08

**Authors:** Shahirah Mansor, Wagee A. Yehye, Azhar Ariffin, Seik Weng Ng

**Affiliations:** a Department of Chemistry, University of Malaya, 50603 Kuala Lumpur, Malaysia

## Abstract

All non-H atoms of the title compound, C_6_H_7_N_3_OS, which exists in the thione form, lie in a common plane (r.m.s. of non-H atoms = 0.08 Å). The amino group of the –NH–NH_2_ substituent forms an intra­molecular hydrogen bond to the S atom. The terminal –NH_2_ group is pyramidally coordinated; it forms a weak N—H⋯O and a weak N—H⋯S hydrogen bond. Furthermore, the N atom is an acceptor for a C—H⋯N contact. The amino group of the ring is a hydrogen-bond donor to the carbonyl O atom of an adjacent mol­ecule, this inter­action giving rise to a linear chain motif running along the *b* axis.

## Related literature

For the synthesis of 3-mercaptonicotinoylhydrazide from 3-mercaptonicotinic acid, see: Katz *et al.* (1958 ▶). For the synthesis of 2-(3,5-di-*tert*-butyl-4-hydroxybenzylsulfanyl)nicotinic acid, see: Mansor *et al.* (2008 ▶).
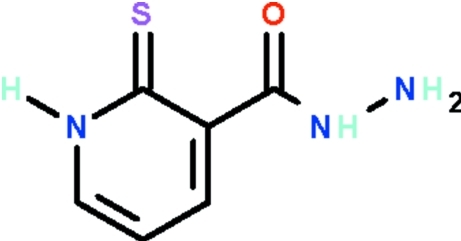


## Experimental

### 

#### Crystal data

C_6_H_7_N_3_OS
*M* *_r_* = 169.21
Triclinic, 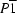

*a* = 7.1952 (2) Å
*b* = 7.4279 (2) Å
*c* = 7.7492 (2) Å
α = 88.205 (2)°
β = 64.201 (2)°
γ = 72.072 (2)°
*V* = 352.22 (2) Å^3^
*Z* = 2
Mo *K*α radiation
μ = 0.40 mm^−1^
*T* = 123 K
0.35 × 0.05 × 0.01 mm

#### Data collection

Bruker SMART APEX diffractometer
Absorption correction: multi-scan (*SADABS*; Sheldrick, 1996 ▶) *T*_min_ = 0.874, *T*_max_ = 0.996
3311 measured reflections
1619 independent reflections
1391 reflections with *I* > 2σ(*I*)
*R*_int_ = 0.015

#### Refinement

*R*[*F* ^2^ > 2σ(*F* ^2^)] = 0.034
*wR*(*F* ^2^) = 0.090
*S* = 1.08
1619 reflections
128 parameters
7 restraints
All H-atom parameters refined
Δρ_max_ = 0.67 e Å^−3^
Δρ_min_ = −0.19 e Å^−3^

### 

Data collection: *APEX2* (Bruker, 2008 ▶); cell refinement: *SAINT* (Bruker, 2008 ▶); data reduction: *SAINT*; program(s) used to solve structure: *SHELXS97* (Sheldrick, 2008 ▶); program(s) used to refine structure: *SHELXL97* (Sheldrick, 2008 ▶); molecular graphics: *X-SEED* (Barbour, 2001 ▶); software used to prepare material for publication: *publCIF* (Westrip, 2010 ▶).

## Supplementary Material

Crystal structure: contains datablocks global, I. DOI: 10.1107/S160053681003521X/bt5335sup1.cif

Structure factors: contains datablocks I. DOI: 10.1107/S160053681003521X/bt5335Isup2.hkl

Additional supplementary materials:  crystallographic information; 3D view; checkCIF report

## Figures and Tables

**Table 1:** Hydrogen-bond geometry (Å, °)

*D*—H⋯*A*	*D*—H	H⋯*A*	*D*⋯*A*	*D*—H⋯*A*
N1—H1⋯O1^i^	0.88 (1)	1.95 (2)	2.751 (2)	152 (2)
N2—H2⋯S1	0.89 (1)	2.24 (2)	3.007 (2)	145 (2)
N3—H3⋯O1^ii^	0.88 (2)	2.36 (3)	3.214 (2)	166 (3)
N3—H4⋯S1^iii^	0.88 (3)	2.85 (3)	3.4173 (18)	124 (2)
C2—H2*A*⋯N3^iv^	0.94 (1)	2.69 (2)	3.323 (4)	125 (2)

Symmetry codes: (i) 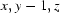; (ii) 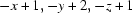; (iii) 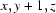; (iv) 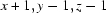.

**Fig. 1.**
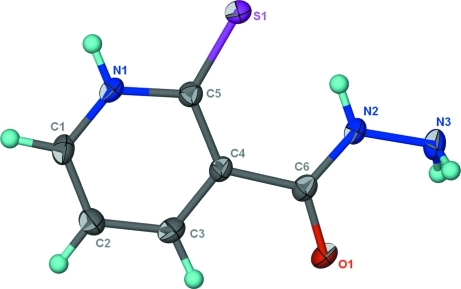
Anisotropic displacement ellipsoid plot (Barbour, 2001) of C6H7N3OS at the 70% probability level; hydrogen atoms are drawn as spheres of arbitrary radius.

**Fig. 2.**
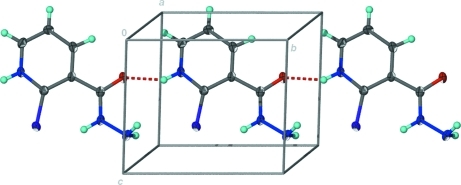
Hydrogen-bonded chain structure.

## supplementary crystallographic information

### Comment

3-Mercaptonicotinylcarbohydrazide is mentioned in the chemical (patent) literature in the context of its synthesis from 3-mercaptonicotinic acid (Katz *et al.*, 1958). This compound was the surprise product of the reaction between ethyl 2-(3,5-di-*tert*-butyl-4-hydroxybenzylsulfanyl)nicotinate and hydrazine. The compound exists in the thione form. The molecule of pryidyl-2(1*H*)-thione-3-carbohydrazide (Scheme I, Fig. 1) is planar (r.m.s. of non-H atoms 0.08 Å). In the six-membered ring, the two carbon–carbon double bonds are regarded as being localized. The amino –NH– group of the –NH–NH_2_ substituent forms an intramolecular hydrogen bond to the double-bond sulfur atom. The terminal –NH_2_ group is pyramidally coordinated; it does not engage in hydrogen bonding. The amino –NH– group of the ring is hydrogen-bond donor to the double-bond oxygen atom an adjacent molecule, this interaction giving rise to a linear chain motif running along the *b*-axis of the triclinic unit cell (Fig. 2).

### Experimental

The synthesis of colorless 2-(3,5-di-*tert*-butyl-4-hydroxybenzylsulfanyl)nicotinic acid was described earlier (Mansor *et al.*, 2008); the acid was first converted to the ethyl ester. The ester (0.80 g, 2 mmol) was dissolved in ethanol (15 ml) and to this was added hydrazine hydrate (0.20 ml, 4 mmol). The mixture was heated for 24 h. The solvent was removed to give a brown gummy solid; this was recrystallized from hexane to afford orange plate-like crystals.

### Refinement

All H-atoms were located in a difference Fourier map, and were refined isotropically with distance restraints of C–H 0.95±0.01 Å and N–H 0.88±0.01 Å.

### Crystal data

**Table:**

C_6_H_7_N_3_OS	*Z* = 2
*M**_r_* = 169.21	*F*(000) = 176
Triclinic, *P*1	*D*_x_ = 1.595 Mg m^−^^3^
Hall symbol: -P 1	Mo *K*α radiation, λ = 0.71073 Å
*a* = 7.1952 (2) Å	Cell parameters from 1745 reflections
*b* = 7.4279 (2) Å	θ = 2.9–28.3°
*c* = 7.7492 (2) Å	µ = 0.40 mm^−^^1^
α = 88.205 (2)°	*T* = 123 K
β = 64.201 (2)°	Plate, orange
γ = 72.072 (2)°	0.35 × 0.05 × 0.01 mm
*V* = 352.22 (2) Å^3^	

### Data collection

**Table:**

Bruker SMART APEX diffractometer	1619 independent reflections
Radiation source: fine-focus sealed tube	1391 reflections with *I* > 2σ(*I*)
graphite	*R*_int_ = 0.015
ω scans	θ_max_ = 27.5°, θ_min_ = 2.9°
Absorption correction: multi-scan (*SADABS*; Sheldrick, 1996)	*h* = −9→9
*T*_min_ = 0.874, *T*_max_ = 0.996	*k* = −9→9
3311 measured reflections	*l* = −10→10

### Refinement

**Table:**

Refinement on *F*^2^	Primary atom site location: structure-invariant direct methods
Least-squares matrix: full	Secondary atom site location: difference Fourier map
*R*[*F*^2^ > 2σ(*F*^2^)] = 0.034	Hydrogen site location: inferred from neighbouring sites
*wR*(*F*^2^) = 0.090	All H-atom parameters refined
*S* = 1.08	*w* = 1/[σ^2^(*F*_o_^2^) + (0.0372*P*)^2^ + 0.3308*P*] where *P* = (*F*_o_^2^ + 2*F*_c_^2^)/3
1619 reflections	(Δ/σ)_max_ = 0.001
128 parameters	Δρ_max_ = 0.67 e Å^−^^3^
7 restraints	Δρ_min_ = −0.19 e Å^−^^3^

### Fractional atomic coordinates and isotropic or equivalent isotropic displacement parameters (Å^2^)

**Table:**

	*x*	*y*	*z*	*U*_iso_*/*U*_eq_	
S1	0.58477 (8)	0.32161 (7)	0.76922 (7)	0.02102 (15)	
O1	0.7744 (2)	0.81569 (18)	0.44198 (19)	0.0210 (3)	
N1	0.8505 (3)	0.1589 (2)	0.4153 (2)	0.0165 (3)	
N2	0.5443 (3)	0.7318 (2)	0.7129 (2)	0.0191 (3)	
N3	0.4375 (3)	0.9211 (2)	0.8063 (2)	0.0218 (4)	
C1	1.0002 (3)	0.1308 (3)	0.2289 (3)	0.0182 (4)	
C2	1.0636 (3)	0.2790 (3)	0.1432 (3)	0.0199 (4)	
C3	0.9635 (3)	0.4575 (3)	0.2526 (3)	0.0181 (4)	
C4	0.8069 (3)	0.4870 (2)	0.4421 (3)	0.0147 (4)	
C5	0.7505 (3)	0.3285 (2)	0.5345 (3)	0.0148 (4)	
C6	0.7070 (3)	0.6906 (2)	0.5353 (3)	0.0160 (4)	
H1	0.805 (4)	0.064 (3)	0.466 (3)	0.036 (7)*	
H2	0.505 (4)	0.638 (3)	0.776 (3)	0.039 (7)*	
H3	0.404 (4)	0.991 (3)	0.724 (3)	0.036 (7)*	
H4	0.533 (3)	0.956 (4)	0.827 (4)	0.032 (7)*	
H1A	1.059 (3)	0.0051 (17)	0.165 (3)	0.020 (5)*	
H2A	1.171 (3)	0.260 (3)	0.0141 (17)	0.027 (6)*	
H3A	1.002 (4)	0.565 (2)	0.199 (3)	0.021 (6)*	

### Atomic displacement parameters (Å^2^)

**Table:**

	*U*^11^	*U*^22^	*U*^33^	*U*^12^	*U*^13^	*U*^23^
S1	0.0251 (3)	0.0148 (2)	0.0172 (2)	−0.00847 (18)	−0.00306 (19)	0.00297 (16)
O1	0.0259 (7)	0.0123 (6)	0.0214 (7)	−0.0081 (5)	−0.0063 (6)	0.0033 (5)
N1	0.0190 (8)	0.0107 (7)	0.0189 (8)	−0.0055 (6)	−0.0072 (6)	0.0025 (6)
N2	0.0210 (8)	0.0102 (7)	0.0194 (8)	−0.0041 (6)	−0.0039 (7)	−0.0003 (6)
N3	0.0250 (9)	0.0114 (7)	0.0216 (8)	−0.0023 (6)	−0.0061 (7)	−0.0017 (6)
C1	0.0199 (9)	0.0125 (8)	0.0192 (9)	−0.0020 (7)	−0.0080 (8)	−0.0016 (7)
C2	0.0196 (9)	0.0176 (9)	0.0161 (9)	−0.0041 (7)	−0.0036 (7)	0.0011 (7)
C3	0.0208 (9)	0.0138 (8)	0.0200 (9)	−0.0065 (7)	−0.0090 (8)	0.0047 (7)
C4	0.0155 (8)	0.0110 (8)	0.0171 (8)	−0.0034 (7)	−0.0075 (7)	0.0016 (6)
C5	0.0143 (8)	0.0137 (8)	0.0160 (8)	−0.0041 (7)	−0.0067 (7)	0.0016 (7)
C6	0.0163 (8)	0.0125 (8)	0.0199 (9)	−0.0041 (7)	−0.0091 (7)	0.0023 (7)

### Geometric parameters (Å, °)

**Table:**

S1—C5	1.696 (2)	N3—H4	0.88 (1)
O1—C6	1.246 (2)	C1—C2	1.365 (3)
N1—C1	1.348 (2)	C1—H1A	0.95 (1)
N1—C5	1.376 (2)	C2—C3	1.395 (3)
N1—H1	0.88 (1)	C2—H2A	0.94 (1)
N2—C6	1.327 (2)	C3—C4	1.380 (3)
N2—N3	1.416 (2)	C3—H3A	0.95 (1)
N2—H2	0.89 (1)	C4—C5	1.433 (2)
N3—H3	0.88 (1)	C4—C6	1.507 (2)
			
C1—N1—C5	125.85 (15)	C3—C2—H2A	121.7 (14)
C1—N1—H1	118.5 (17)	C4—C3—C2	122.34 (17)
C5—N1—H1	115.6 (17)	C4—C3—H3A	116.8 (14)
C6—N2—N3	121.63 (15)	C2—C3—H3A	120.9 (14)
C6—N2—H2	119.0 (17)	C3—C4—C5	119.45 (16)
N3—N2—H2	119.3 (17)	C3—C4—C6	115.35 (15)
N2—N3—H3	106.3 (17)	C5—C4—C6	125.20 (16)
N2—N3—H4	107.0 (17)	N1—C5—C4	114.58 (15)
H3—N3—H4	109 (2)	N1—C5—S1	116.57 (13)
N1—C1—C2	119.76 (16)	C4—C5—S1	128.82 (14)
N1—C1—H1A	116.8 (14)	O1—C6—N2	121.97 (16)
C2—C1—H1A	123.4 (14)	O1—C6—C4	119.33 (16)
C1—C2—C3	117.83 (17)	N2—C6—C4	118.66 (15)
C1—C2—H2A	120.5 (14)		
			
C5—N1—C1—C2	−0.3 (3)	C3—C4—C5—S1	173.23 (15)
N1—C1—C2—C3	−2.1 (3)	C6—C4—C5—S1	−7.1 (3)
C1—C2—C3—C4	0.7 (3)	N3—N2—C6—O1	0.2 (3)
C2—C3—C4—C5	3.0 (3)	N3—N2—C6—C4	−177.57 (17)
C2—C3—C4—C6	−176.77 (17)	C3—C4—C6—O1	−2.5 (3)
C1—N1—C5—C4	3.8 (3)	C5—C4—C6—O1	177.77 (17)
C1—N1—C5—S1	−174.60 (15)	C3—C4—C6—N2	175.28 (17)
C3—C4—C5—N1	−4.9 (3)	C5—C4—C6—N2	−4.4 (3)
C6—C4—C5—N1	174.76 (16)		

### Hydrogen-bond geometry (Å, °)

**Table:**

*D*—H···*A*	*D*—H	H···*A*	*D*···*A*	*D*—H···*A*
N1—H1···O1^i^	0.88 (1)	1.95 (2)	2.751 (2)	152 (2)
N2—H2···S1	0.89 (1)	2.24 (2)	3.007 (2)	145 (2)
N3—H3···O1^ii^	0.88 (2)	2.36 (3)	3.214 (2)	166 (3)
N3—H4···S1^iii^	0.88 (3)	2.85 (3)	3.4173 (18)	124 (2)
C2—H2A···N3^iv^	0.94 (1)	2.69 (2)	3.323 (4)	125 (2)

Symmetry codes: (i) *x*, *y*−1, *z*; (ii) −*x*+1, −*y*+2, −*z*+1; (iii) *x*, *y*+1, *z*; (iv) *x*+1, *y*−1, *z*−1.

## References

[bb1] Barbour, L. J. (2001). *J. Supramol. Chem.* **1**, 189–191.

[bb2] Bruker (2008). *APEX2* and *SAINT* Bruker AXS Inc., Madison, Wisconsin, USA.

[bb3] Katz, L., Cohen, M. S. & Schröder, W. (1958). US Patent 282487.

[bb4] Mansor, S., Yehye, W. A., Ariffin, A., Rahman, N. A. & Ng, S. W. (2008). *Acta Cryst.* E**64**, o1778. 10.1107/S1600536808026056 PMC2960523 21201758

[bb5] Sheldrick, G. M. (1996). *SADABS* University of Göttingen, Germany.

[bb6] Sheldrick, G. M. (2008). *Acta Cryst.* A**64**, 112–122. 10.1107/S0108767307043930 18156677

[bb7] Westrip, S. P. (2010). *J. Appl. Cryst.* **43**, 920–925.

